# The potential of *Olea europaea* extracts to prevent TGFβ1-induced epithelial to mesenchymal transition in human nasal respiratory epithelial cells

**DOI:** 10.1186/s12906-018-2250-5

**Published:** 2018-06-26

**Authors:** Rabiatul Adawiyah Razali, Nik Ahmad Hafiz Nik Ahmad Eid, Turkambigai Jayaraman, Muhammad Asyrafi Amir Hassan, Nabilah Qistina Azlan, Nur Farhana Ismail, Nur Qisya Afifah Veronica Sainik, Muhammad Dain Yazid, Yogeswaran Lokanathan, Aminuddin Bin Saim, Ruszymah Bt Hj Idrus

**Affiliations:** 1 Department of Physiology, Faculty of Medicine, Universiti Kebangsaan Malaysia Medical Centre, Universiti Kebangsaan Malaysia, Cheras, 56000 Kuala Lumpur, Malaysia; 2 Tissue Engineering Centre, Faculty of Medicine, Universiti Kebangsaan Malaysia Medical Centre, Universiti Kebangsaan Malaysia, Cheras, 56000 Kuala Lumpur, Malaysia; 3 Ear, Nose & Throat Consultant Clinic, Ampang Puteri Specialist Hospital, 68000 Ampang, Selangor Malaysia

**Keywords:** Olive, TGFβ1, EMT, Vimentin, Circularity, E-cadherin

## Abstract

**Background:**

One of the molecular mechanisms involved in upper airway-related diseases is epithelial-to-mesenchymal transition (EMT). *Olea europaea* (OE) has anti-inflammatory properties and thus, great potential to prevent EMT. This study aimed to investigate the effect of OE on EMT in primary nasal human respiratory epithelial cells (RECs).

**Methods:**

Respiratory epithelial cells were isolated and divided into four groups: control (untreated), treated with 0.05% OE (OE group), EMT induced with 5 ng/ml of transforming growth factor beta-1 (TGFβ1 group) and treated with 5 ng/ml TGFβ1 + 0.05% OE (TGFβ1 + OE group). The effects of OE treatment on growth kinetics, morphology and protein expression in RECs were evaluated. Immunocytochemistry analysis was performed to quantitate the total percentage of E-cadherin and vimentin expression from day 1 to day 3.

**Results:**

There were no significant differences between untreated RECs and OE-treated RECs in terms of their morphology, growth kinetics and protein expression. Induction with TGFβ1 caused RECs to have an elongated spindle shape, a slower proliferation rate, a higher expression of vimentin and a lower expression of E-cadherin compared with the control. Cells in the TGFβ1 + OE group had similar epithelial shape to untreated group however it had no significant differences in their proliferation rate when compared to TGFβ1-induced RECs. Cells treated with TGFβ1 + OE showed significantly reduced expression of vimentin and increased expression of E-cadherin compared with the TGFβ1 group (*P* < 0.05).

**Conclusion:**

The ability of OE to inhibit EMT in RECs was shown by TGFb1-induced EMT REC morphology, growth kinetics and protein expression markers (E-cadherin and vimentin) upon treatment with OE and TGFβ1. Therefore, this study could provide insight into the therapeutic potential of OE to inhibit pathological tissue remodelling and persistent inflammation.

## Background

Chronic rhinosinusitis is a pathological disease due to inflammation of the sinonasal tract caused by infection or trauma. This long-term chronic inflammation will cause pathological changes in the mucosal epithelial layer, thus causing sinus ostium obstruction and symptoms such as nasal discharge, facial pain and even reduction of olfactory function [1–3]. This debilitating disease affects about 10.9% of the world’s population [3] and significantly reduces the patient’s quality of life [4]. The first line of treatment is usually antibiotics and a nasal decongestant with or without steroids. Failure of such treatment or the presence of obvious anatomical obstruction will eventually lead to sinus surgery to clear the obstruction and remove the diseased mucosal epithelium [5].

Several factors that could contribute to the pathogenesis of chronic rhinosinusitis are anatomic factors, allergies and viral or bacterial infections [2]. Respiratory epithelial cells (RECs) line the nasal airway and have an important function in mediating innate immunity to fight allergens and pathogens such as bacteria, viruses and fungi. Unfortunately, prolonged exposure and reaction to these pathological agents may lead to chronic inflammation and tissue remodelling.

Tissue remodelling is an important component of the response to inflammatory insult. It is a dynamic process involving dedifferentiation of epithelial cells, increased matrix production, degradation and subepithelial base membrane thickening [5, 6]. Differentiation of epithelial cells occurs when the cells change their phenotype from epithelial to mesenchymal through a normal reversible process called epithelial-to-mesenchymal transition (EMT) [6]. Epithelial cells will start to lose their typical morphology, polarity and junctional attachments and become motile. These occurrences are indicated by downregulation of expression of junctional proteins such as E-cadherin, ZO-1, claudins and occludin [6, 7]. Additionally, these epithelial cells now acquire mesenchymal phenotypes such as expression of vimentin and α-SMA due to rearrangement of cytoskeletal filaments that will prepare them to migrate.

There are three types of EMT [8, 9]. Type 1 EMT was associated with the primary mesenchymal cells going through mesenchymal–epithelial transition (MET) to form secondary epithelia which occurred during embryogenesis. Type 2 EMT happened during wound healing and organ regeneration which would help in tissue reconstruction. However, prolonged inflammation and insults will lead to fibrosis that was associated with Type 2 EMT. Type 3 EMT is associated with cancer progression and metastasis.

Insults to epithelial layer may cause injury or wound which will be repaired by the wound healing mechanisms and EMT. Epithelial cells undergoing EMT will become enlarged and migrate across the wound bed before they divide and proliferate to form a new epidermis layer [10, 11].

However, prolonged inflammation will cause alterations in the composition and organization of an organ due to continual EMT remodeling process, which eventually will lead to fibrogenesis [5, 11]. Type 2 EMT has been associated with myofibroblast that expresses α-SMA. This EMT epithelial cells or myofibroblasts travel through the basement membrane and accumulate at the tissue interstitium where they will synthesize matrix and collagens [11]. The dysregulation of injury-triggered EMT is believed to contribute to fibrosis of multiple organs, including those of the respiratory system [11].

Various growth and differentiation factors can induce or regulate EMT, including TGF-β, fibroblast growth factor, hepatic growth factor, platelet-derived growth factor and Wnt and Notch proteins [12]. However, TGFβ1 has been regarded as the most important growth factor for EMT due to its correlation with the extent of fibrosis and myofibroblast-like cell induction [13]. Upon TGFβ1 treatment, epithelial cells change their morphology from cuboidal to elongated spindle shaped; this is accompanied by increased cell motility, decreased expression of epithelial markers and increased expression of mesenchymal markers such as vimentin [5, 14, 15].

In chronic rhinosinusitis, the epithelial layer shows a characteristic goblet cell hyperplasia, subepithelial oedema, inflammatory cell infiltration and fibrosis [16]. These would cause further epithelial damage, such as shedding and basement membrane thickening, which can cause physical and functional barrier defects [5, 6]. Many studies have confirmed the loss of epithelial markers (e.g. E-cadherin) and gain of mesenchymal markers (e.g. vimentin) in chronic rhinosinusitis epithelial layers and the pathogenesis of nasal polyps, which shows that there are correlations between EMT and chronic rhinosinusitis with or without nasal polyps [2, 7, 17]. Therefore, one preventive method is to modulate EMT such that it does not cause severe tissue remodelling and persistent inflammation.

Several studies have demonstrated the ability of natural products to modulate EMT [18, 19]. *Olea europaea*, commonly known as olive, is a promising natural product with proven health benefits due to the presence of phenolics and flavonoids [20, 21]. Olive is the fruit from the olive tree (*Olea europaea L*), which belongs to the family Oleaceae and is commonly included in the diet of Mediterranean people. The major phenolic compounds that have been identified and quantified in olive oil belong to three different classes: simple phenols (hydroxytyrosol, tyrosol); secoiridoids (oleuropein, the aglycone of ligstroside, and their respective decarboxylated dialdehyde derivatives) and lignans [(+)-1-acetoxypinoresinol and (+)- pinoresinol] [22].

Olive has been proven to have various benefits to consumers, including the ability to alter the structure of neurotoxic proteins that are believed to contribute to the debilitating effects of Alzheimer’s disease [23] and the ability to impede and attenuate cell proliferation, invasiveness, fibrogenesis and EMT in breast cancer cell, kidney cells, and prostate cancers [24–26]. Besides that, olive also has been used traditionally in treating asthma, haemorrhoids, intestinal diseases and in reducing blood sugar and cholesterol [27]. However, no study on the effect of olive extract or its bioactive compound on EMT in nasal respiratory epithelial cells has been conducted.

Therefore, this study aimed to investigate the effects of olive extract on TGFβ1-induced EMT in human respiratory epithelial cells.

## Methods

This study was approved by Universiti Kebangsaan Malaysia Research Ethics Committee (FF-2017-363).

### Human nasal REC isolation

Redundant human nasal turbinate tissue was obtained with a written consent from four Asian patients who had undergone a turbinectomy procedure. Turbinate tissue was then washed with Dulbecco’s Phosphate-Buffered Saline (DPBS) (Gibco, USA) to remove blood and mucus. Next, the epithelial layer was separated from the tissue and minced completely before being digested in 0.6% Collagenase Type I (Worthington, USA) for 60 min in a shaker incubator at 37 °C. After the tissue was fully digested, it was centrifuged for 5 min at 2370×*g*. Then, the supernatant was discarded and the pellet washed with DPBS and re-centrifuged for 5 min at 2370×*g* .

The cell pellets were suspended in growth medium consisting of Airway Epithelial Growth Medium (AEGM) (PromoCell, USA), Defined Keratinocyte Serum-Free Medium (DKSFM) (GIBCO, USA) and Dulbecco’s Modified Eagle Medium: Nutrient Mixture F-12 (F12: DMEM) supplemented with 5% Fetal Bovine Serum (FBS) (BioWest, USA), in a 1:1:2 ratio and seeded into a 6-well plate (Thermo Fischer, USA). All cells were then cultured at 37 °C in 5% CO2 incubator.

The medium was changed every 2 days until it reached 80–90% confluency. Subsequently, the co-culture of RECs and fibroblast was differentially trypsinized to remove fibroblasts from the culture plate. The medium was changed every 2 days until the cells reached 80–90% confluency before being trypsinized into passage 1 (P1) or passage 2 (P2), which were used as the experimental passages.

### Cytotoxicity assay

The cytotoxic effect of commercially available *Olea europaea* (OE) extract (Olivenol plus+, CreAgri Inc., USA) was evaluated using the Vybrant™ MTT (3-(4,5-Dimethylthiazol-2-yl)-2,5-Diphenyltetrazolium Bromide) cell proliferation assay kit (Invitrogen, USA) following the manufacturer recommendations. Briefly, respiratory epithelial cells at passage 1 were grown in a 48-well plate and treated with several concentrations of OE extracts (0, 0.025, 0.05, 0.1, 0.15, 0.2, 0.4. 1 and 2% (*v*/v) for 24 h. Subsequently, OE-treated RECs medium were removed and replaced with 200 μl of fresh medium. About 20 μl of MTT solution were added to the well and incubated for 4 h at 37 °C before 200 μl of sodium dodecyl sulphate–hydrogen chloride (SDS-HCL) solution was added and were further incubated for another 4 h. All of the solution in the well were divided into two replicates and about 210 μl of solution was transferred into 1 well of 96-well plates before the absorbance was read at 570 nm. Four samples were assayed in triplicate for this experiment. One optimum concentration was chosen to be used for further experimentation.

### Quantification of total cell attached

Respiratory epithelial cells were observed 24 h after TGFβ1 induction. In order to profile the growth of TGFβ1 induced RECs, about five independent fields of REC images were captured [28]. The total number of cells attached to the surface was calculated and quantitated following the equation below:

$$ \mathrm{Total}\kern0.17em \mathrm{cell}\mathrm{s}\kern0.17em \mathrm{attached}=\frac{\mathrm{Average}\kern0.17em \mathrm{cell}\kern0.17em \mathrm{count}}{\mathrm{Objective}\kern0.17em \mathrm{area}\kern0.17em \mathrm{of}\kern0.17em \mathrm{the}\kern0.17em \mathrm{microscope}} $$

### Immunocytochemical analysis

Expression levels of E-cadherin and vimentin were evaluated using immunocytochemical analysis [25]. Cells were washed with DPBS, fixed with 4% paraformaldehyde (PFA) for 30 min (Sigma-Aldrich, USA), permeabilized for 20 min with 0.5% Triton X-100 solution (Sigma-Aldrich) and then blocked with 10% goat serum for 1 h at 37 °C. The cells were then incubated with 1:200 mouse anti-E-cadherin antibody (ab1416) and 1:2000 rabbit anti-Vimentin antibody (ab92547) (Abcam, USA) overnight at 4 °C. On the following day, the cells were washed before being incubated with 1:300 diluted Alexa Fluor 594 anti-rabbit IgG (Invitrogen, USA) and Alexa Fluor 488 anti-mouse (Invitrogen) for 1 h at 37 °C. Nuclei were counterstained with DAPI. Fluorescence images were captured with a Nikon Eclipse Ti fluorescence microscope (Nikon, Japan). The total number of cells expressing vimentin was calculated from five independent fields of images following the same equation above.

### *Olea europaea* supplementation

Passage 2 RECs at about 40% confluency were seeded into four different conditions, namely control (untreated RECs only), 0.05% OE (RECs treated with *Olea europaea* extract) (CreAgri Inc., USA), TGFβ1 (RECs induced with 5 ng/ml TGFβ1) and TGFβ1 + OE (RECs treated with 0.05% *Olea europaea* extract and induced with 5 ng/ml TGFβ1). All control and treatment groups were subjected to morphological assessment [29], growth kinetic quantification [28] and immunocytochemical analysis [25].

### Morphology assessment

Morphological observation of RECs was performed for 72 h. Images were captured daily with a Nikon A1R Confocal microscope (Nikon, Japan). Cell circularity was quantitated using ImageJ (version 1.51j8, National Institutes of Health, USA) at day 3 of induction. Cellular morphology was assessed based on the value of circularity where a value approaching 0 indicated an elongated shape, and as the value increased to 1, the shape became more circular.

### Cell proliferation and growth kinetics

About five independent fields of REC images were captured at 0, 24, 48 and 72 h. Total numbers of cells attached at the aforementioned time points were calculated. The proliferation rate and population doubling time (PDL) were calculated using the following formula:

$$ \mathrm{Proliferation}\ \mathrm{rate}\ {\left(\mathrm{h}\right)}^{-1}=\frac{\ln \Big(\mathrm{Total}\ \mathrm{cells}\ \mathrm{attached}\ \left(\mathrm{final}\right)/\mathrm{Total}\ \mathrm{cells}\ \mathrm{attached}\ \left(\mathrm{initial}\right)}{\mathrm{Time}} $$

$$ \mathrm{PDL}=\frac{\left(\mathrm{Time}\ \mathrm{x}\log\ (2)\right)}{\log \Big(\mathrm{total}\ \mathrm{cells}\ \mathrm{attached}\ \left(\mathrm{final}\right)-\log\ \left(\mathrm{total}\ \mathrm{cells}\ \mathrm{attached}\ \left(\mathrm{initial}\right)\right)} $$

### Statistical analysis

Experiments were performed in triplicate and repeated on at least three biological samples (*n* = 3), and data are presented as mean ± SEM. For statistical analysis, ANOVA was used. Statistical analysis was performed using Prism Version 7.0 software (GraphPad, Software Inc., USA). Results were considered statistically significant at *P* < 0.05. All values are expressed as mean ± SEM.

## Results

### OE increases REC proliferation

**Fig. 1:**
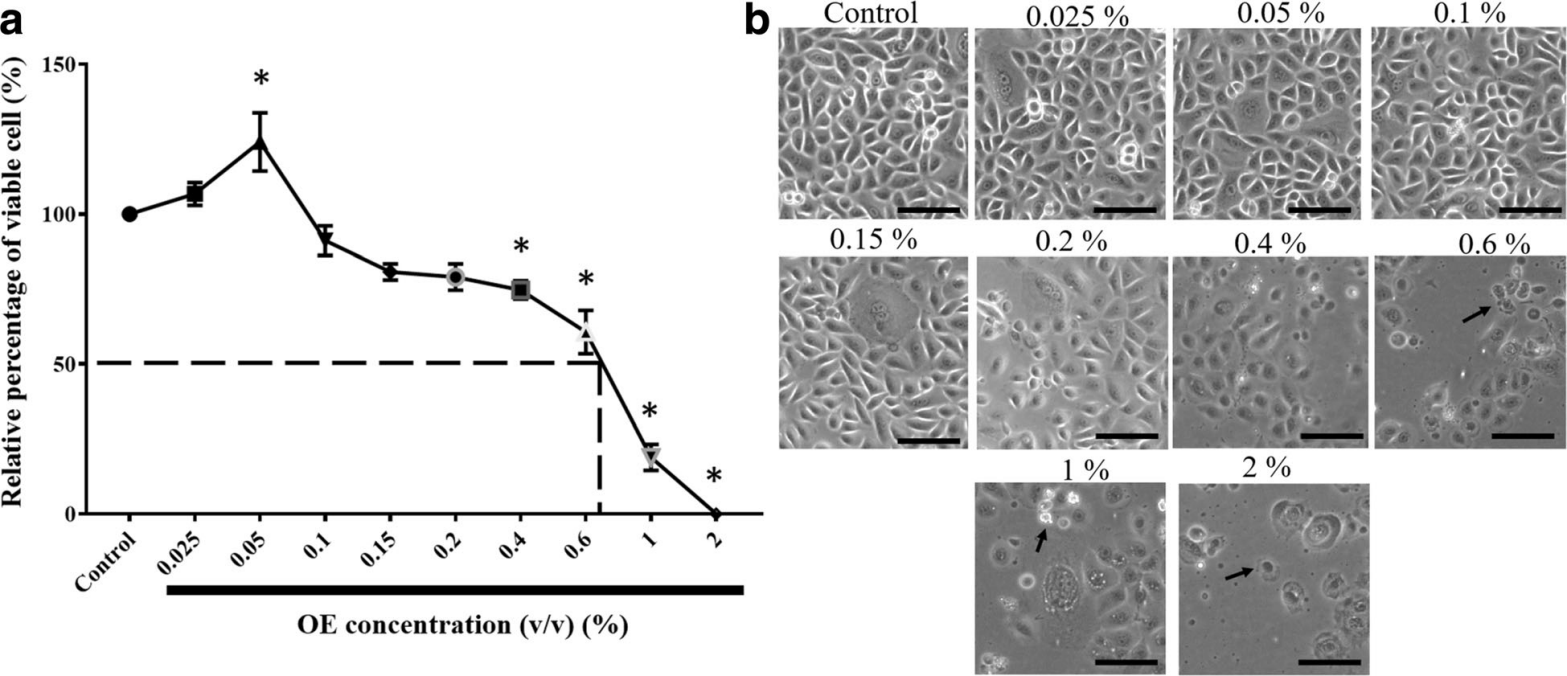
**a** Cytotoxicity of OE determined by MTT assay. *P < 0.05: significantly different from control. **b** Morphology of RECs treated with several concentrations of OE for 24 h (arrow showing round and shrunken cells)

The MTT assay was performed to examine the effects of OE on normal human RECs. Treatment with 0.05% OE was found to significantly increase the total number of viable RECs by 24% compared with the control. However, there was a significant concentration-dependent inhibition of cell proliferation starting from 0.4% OE, with an inhibitory concentration 50 (IC_50_) of 0.69% (Fig. 1a). Moreover, as the percentage of OE increased above 0.6% (*v*/v), RECs started to become rounded and shrunken (Fig. 1b). This assay also showed that 0.05% OE has a positive effect on cell viability and proliferation of RECs. Therefore, it was chosen as the selected concentration to be used throughout the experiment.

### TGFβ1 causes primary RECs to undergo EMT

**Fig. 2:**
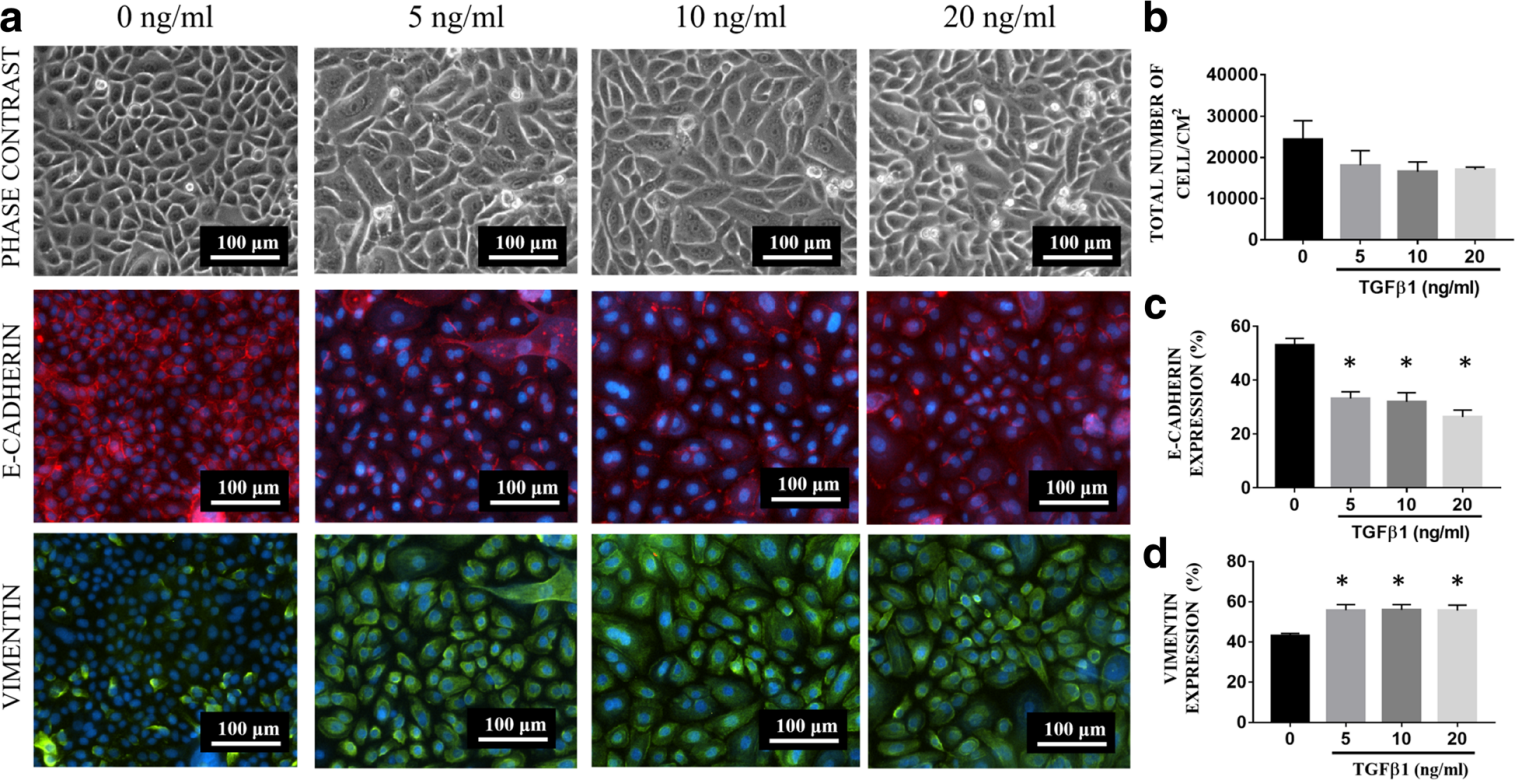
**a** Phase-contrast and fluorescence microscope image of RECs treated with 0, 5, 10 and 20 ng/ml of TGFβ1. **b** Total number of cells after a 24-h treatment with 0, 5, 10 and 20 ng/ml. There is a reduction in the total number of cells after 24 h of treatment in all the experimental groups compared with the control (0 ng/ml). **c** Vimentin expression. **d** E-cadherin expression. *P < 0.05 indicates a significant difference compared with the control

In order to develop an EMT model, three concentrations of TGFβ1 were used to evaluate both epithelial and mesenchymal expression markers (Fig. 2). After induction of RECs with TGFβ1 for 24 h, all RECs that had undergone TGFβ1-induced EMT appeared larger and elongated, similar in shape to mesenchymal cells. Moreover, RECs in the 5, 10, and 20 ng/ml TGFβ1-induced EMT group were lower in number (1.8 × 10^4^ ± 6.2 × 10^3^, 1.7 × 10^4^ ± 4.7 × 10^3^, 1.7 × 10^4^ ± 1.2 × 10^3^ cells/cm^2^ respectively) than those in the untreated group (2.4 × 10^4^ ± 9.1x10^3^cells/cm^2^). Immunocytochemical analysis of E-cadherin and vimentin showed that, upon treatment with 5, 10, and 20 ng/ml of TGFβ1, E-cadherin expression was significantly lower (33.2% ± 2.5, 32% ± 3.4 and 26.4% ± 2.5 respectively), whereas vimentin expression was significantly higher (55.8% ± 2.8, 56% ± 2.6, and 55.6% ± 2.8 respectively), compared with untreated RECs (E-cadherin:53% ± 2.5 and vimentin: 43% ± 1.2). As induction with 5 ng/ml TGFβ1 could induce RECs to undergo EMT, this concentration was chosen to be used throughout this experiment.

### OE increases the circularity of RECs with TGFβ1-induced EMT

**Fig. 3:**
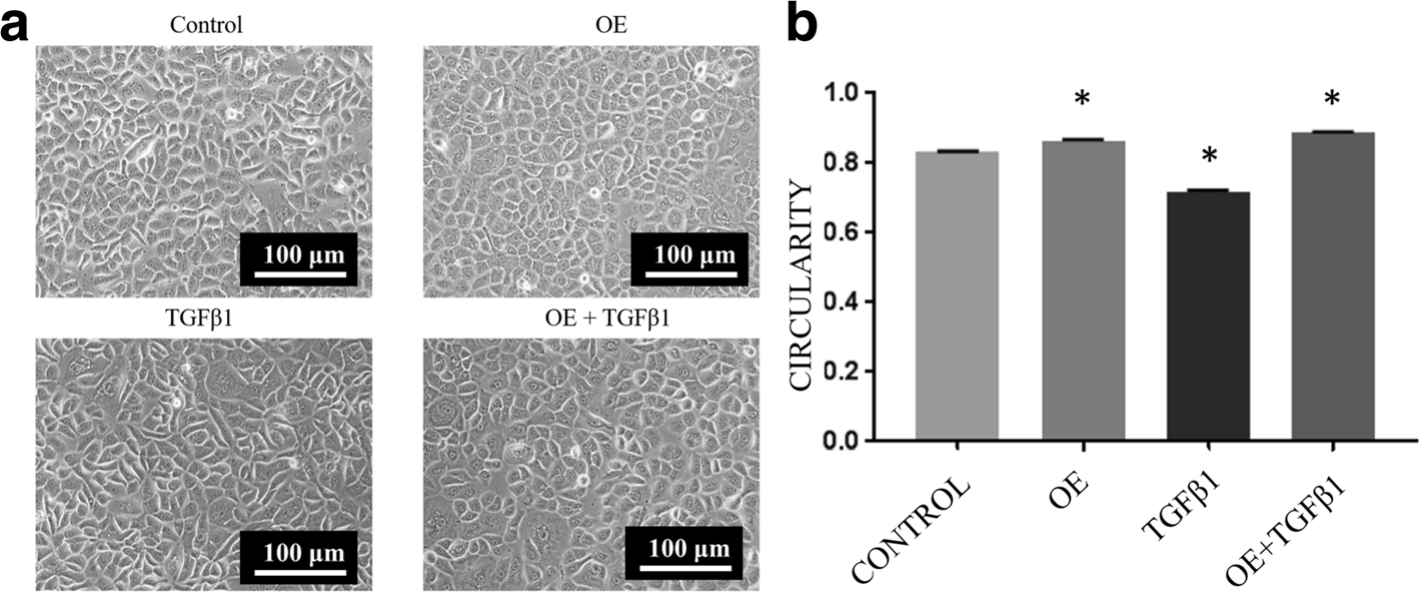
**a** Morphology of untreated RECs (control), 0.05% OE-treated RECs (OE), RECs with EMT induced by 5 ng/ml TGFβ1 (TGFβ1) and RECs with EMT induced by TGFβ1 treated with OE (TGFβ1 + OE) on day 3. The TGFβ1 group displays a more elongated structure than the OE and TGFβ1 + OE groups. **b** Cell circularity as analyzed using ImageJ software. *P < 0.05 indicates significant difference compared with control

The morphology of treated and untreated RECs was observed, and circularity analysis was performed to quantify morphological changes. Respiratory epithelial cells treated with 0.05% OE were more circular (0.865 ± 0.002) than control RECs (0.832 ± 0.002). However, upon treatment with 5 ng/ml of TGFβ1, RECs were found to become spindle-shaped (0.717 ± 0.005), resembling mesenchymal cells. Interestingly, RECs treated with TGFβ1 and OE were found to be more circular (0.886 ± 0.002) than control cells and TGFβ1-induced RECs without OE (EMT RECs) (Fig. 3).

### TGFβ1-induced REC proliferation is not affected by OE treatment

**Fig. 4:**
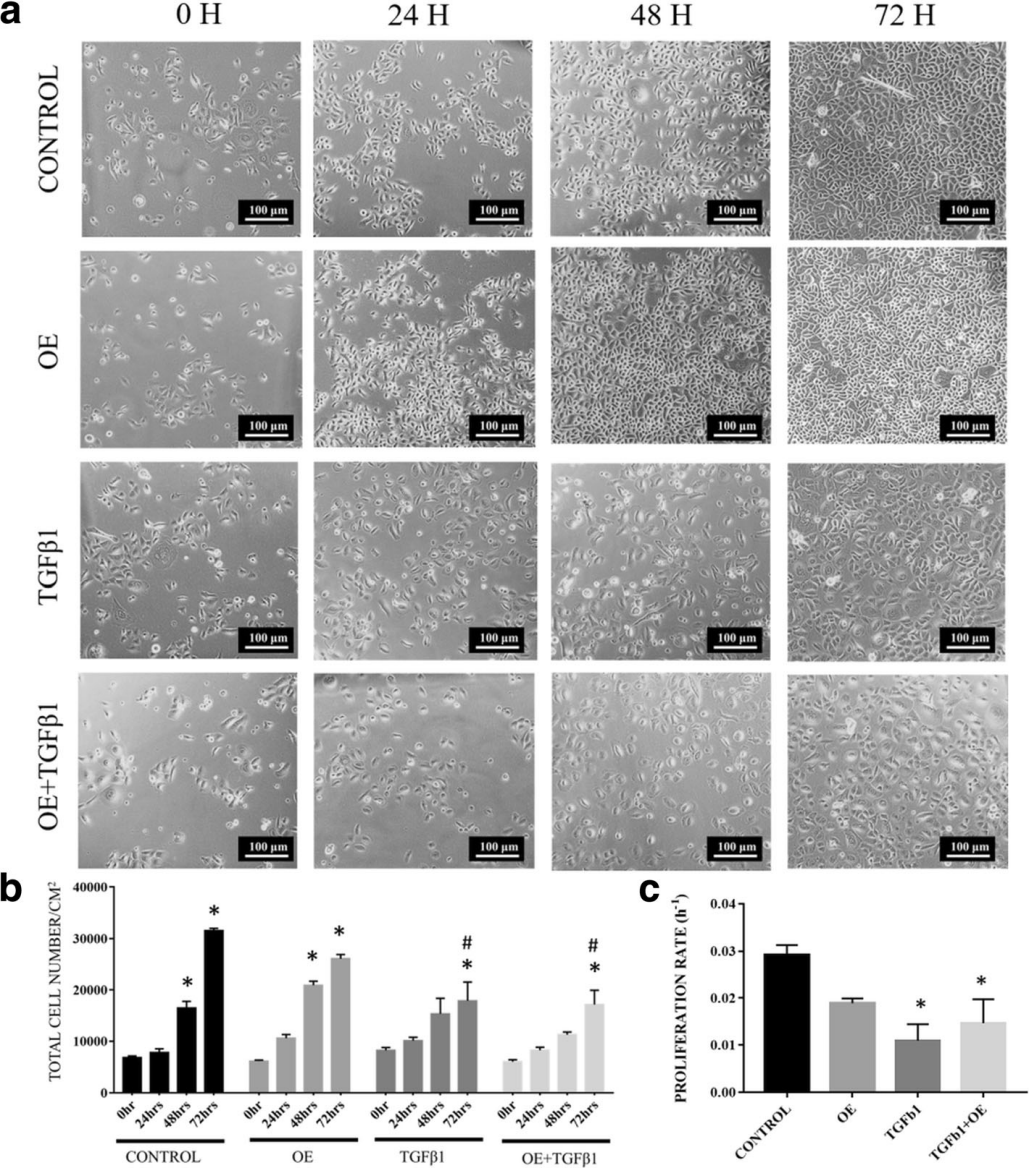
**a** Distribution of untreated RECs (control), 0.05% (*v*/v) OE-treated RECs (OE), RECs induced to EMT with 5 ng/ml TGFβ1 (TGFβ1) and RECs induced to EMT with TGFβ1 and treated with OE (TGFβ1 + OE) from 0 h to 72 h. **b** Total cell number/cm^2^ versus (vs) time per group. *P < 0.05 indicates significant difference when compared with 0 h and # *P* < 0.05 indicates significant difference when compared with the control group. **c** The proliferation rate of cells. *P < 0.05 indicates significant difference when compared with the control

There was a significant increase in the total cell number of untreated RECs and in the OE-treated group at 48 h and 72 h compared with 0 h. The TGFβ1 and TGFβ1 + OE groups showed a significant increase in total cell number only after 72 h (Fig. 4b). The untreated REC group had a higher proliferation rate (0.03 h^− 1^ ± 0.002) than the OE, TGFβ1 + OE and TGFβ1 groups, with a population doubling time (PDL) of 24 h ± 2 h. This was followed by the OE group (0.019 h^− 1^ ± 0.001), with a PDL of 37 h ± 2 h (Fig. 4c). However, the proliferation rates in the TGFβ1 group and the TGFβ1 + OE group were significantly lower compared with the control (0.011 h^− 1^ ± 0.003 and 0.015 h^− 1^ ± 0.005, respectively) with PDL of 91 h ± 42 h and 72 h ± 37 h, respectively.

### OE reduces vimentin expression in TGFβ1-induced RECs

**Fig. 5:**
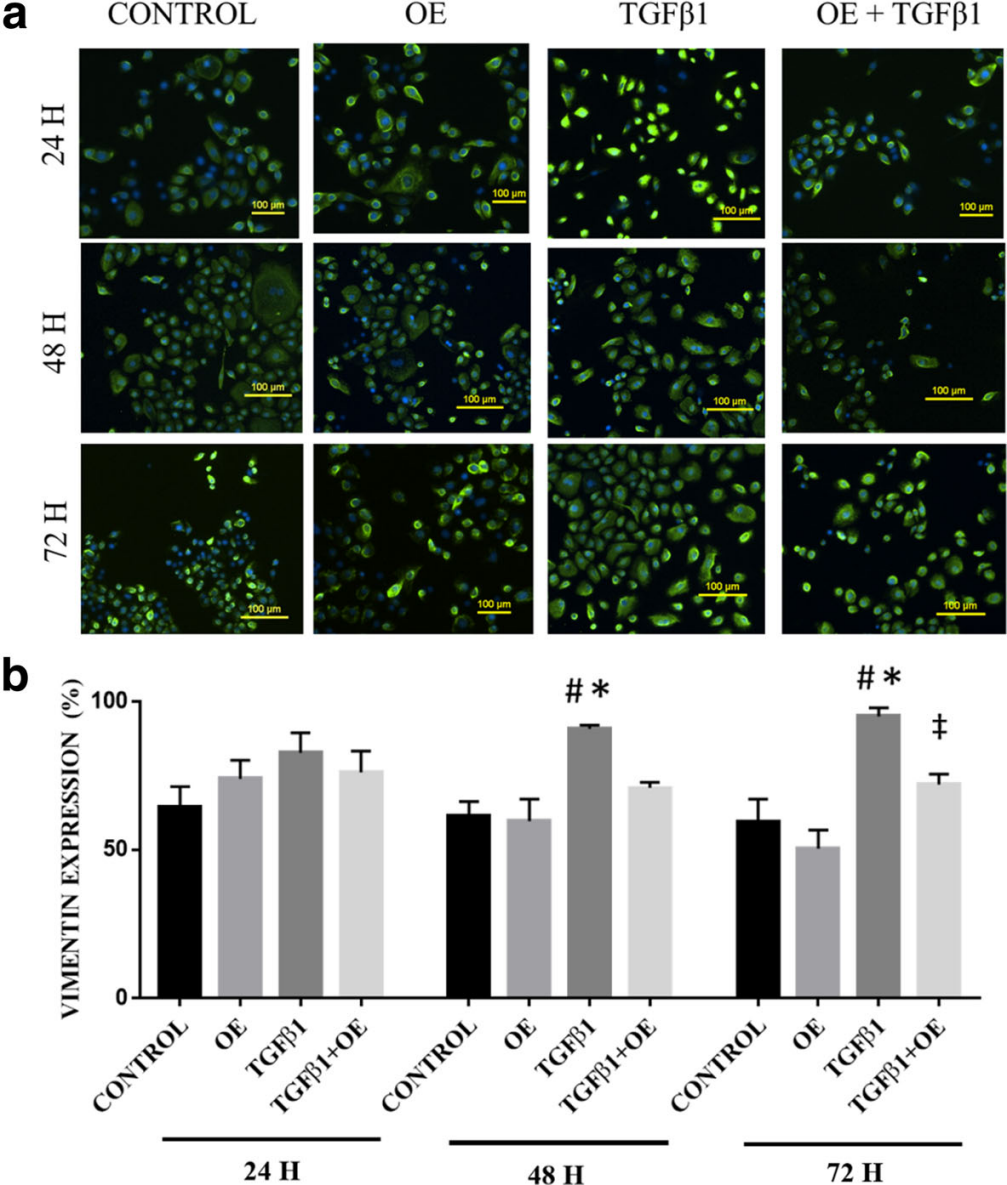
**a** Vimentin expression in untreated RECs (control), 0.05% OE-treated RECs (OE), RECs induced to EMT with 5 ng/ml TGFβ1 (TGFβ1) and RECs induced to EMT with TGFβ1 and treated with OE (TGFβ1 + OE) from 0 h until 72 h. Green cytoplasm indicates vimentin expression, and blue is DAPI-stained nuclei. **b** Vimentin expression (%) vs time per group. *P < 0.05 indicates significant differences when compared with the control, #*P* < 0.05 indicates significant differences when compared with OE, ‡*P* < 0.05 indicates significant differences when compared with TGFβ1

Figure 5 shows the expression of vimentin in each treatment group. At 24 h, there were no significant differences in vimentin expression between all tested groups. However, at 48 h TGFβ1 was found to significantly increase vimentin expression by RECs (90.81% ± 1.27) compared with the control (61.18% ± 5.05) and OE groups (59.59% ± 7.49) (Fig. 5b). After 72 h, a significant 22% reduction in vimentin expression was seen in the TGFβ1 + OE group (72.00% ± 3.54) compared with TGFβ1-induced RECs (94.95% ± 2.94). There were no significant differences in vimentin expression in RECs treated with OE compared with untreated RECs (control).

### OE increases E-cadherin expression in TGFβ1-induced RECs

**Fig. 6:**
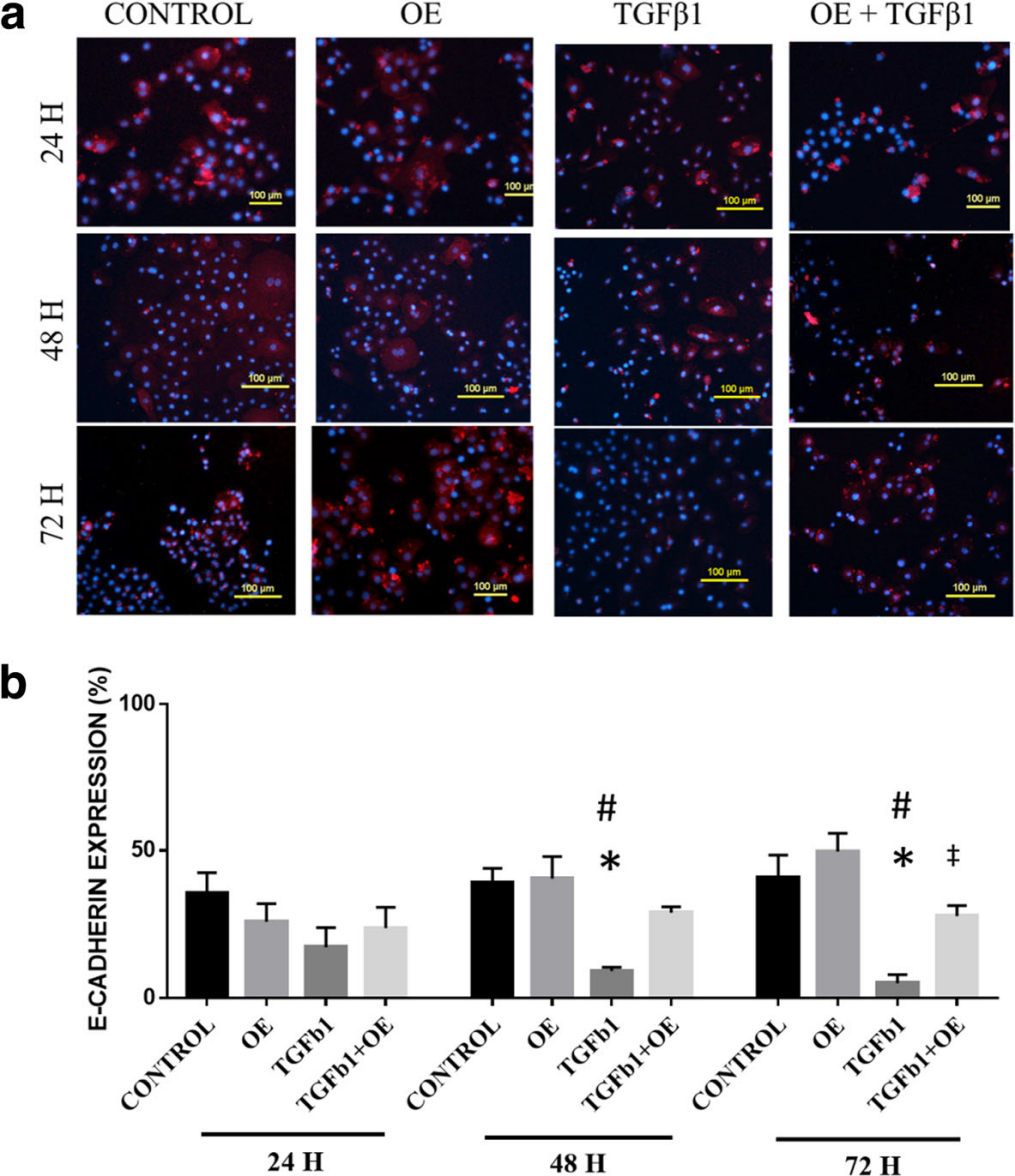
**a** E-cadherin expression in untreated RECs (control), 0.05% OE-treated RECs (OE), RECs induced to EMT with 5 ng/ml TGFβ1 (TGFβ1) and RECs induced to EMT with TGFβ1 and treated with OE (TGFβ1 + OE) from 0 h until 72 h. Red colour indicates E-cadherin expression, and blue is the nucleus. **b** E-cadherin expression (%) at various time points. *P < 0.05 indicates significant differences when compared with the control, #*P* < 0.05 indicates significant differences when compared with OE, ‡*P* < 0.05 indicates significant differences when compared with TGFβ1

After a 24 h incubation, there were no significant differences in E-cadherin expression between all tested groups. Respiratory epithelial cells that underwent TGFβ1-induced EMT had lower E-cadherin expression after 48 h (9.19% ± 1.27) of TGFβ1 treatment compared with untreated RECs (38.82% ± 5.05) and the OE group (40.41% ± 7.49) (Fig. 6). There were no significant differences in E-cadherin expression in the TGFβ1 + OE group compared with that of untreated RECs and the OE group at 24 h and 48 h. However, at 72 h, E-cadherin expression was higher in the TGFβ1 + OE group (27.99% ± 3.54) compared with TGFβ1-induced RECs (5.05% ± 2.94).

## Discussion

Persistent injury and inflammation to the respiratory epithelial layer due to infection or allergens can cause fibrosis, thickening of the subepithelial membrane and an impaired nasal epithelial barrier. These alterations are known to cause damage to the nasal epithelium and contribute to nasal polyp formation [25]. Changes that occur during insults can be marked by the activation of EMT, where the epithelial cells lose their epithelial phenotype and become mesenchymal cells, which can lead to changes in epithelial morphology. This occurrence can be investigated by observing the decreasing expression of epithelial protein markers such as E-cadherin and ZO-1 and increasing expression of mesenchymal markers such as vimentin. E-cadherin is an important component for epithelial adherence junction. Reduction in the expression of E-cadherin protein marked the initiation of EMT where cells detached from one another and started the migration process [30]. Meanwhile, vimentin is a critical mesenchymal markers that are highly elevated during EMT which indicated changes in phenotype of cells from epithelial to mesenchymal [8]. In this study, we examined the potential of *Olea europaea* extract (OE) to prevent TGFβ1-induced EMT in human RECs.

We have observed concentration-dependent effects of OE on RECs, in which 0.05% (*v*/v) OE increased REC proliferation. Reduction of proliferation of RECs began at 0.4% (v/v) OE, indicating that RECs were undergoing apoptosis (IC_50_ = 0.69%). Therefore, in this study, we used OE at a concentration of 0.05%. Olive at suitable concentrations has been observed to have selective activity whereby it increases proliferation of normal non-disease cells while causing death in diseased cells [31, 32].

Treatment with OE did not cause any changes in RECs in terms of protein expression and morphology. Interestingly, OE maintained the cuboidal shape of RECs, while retaining higher expression of E-cadherin and lower vimentin expression, similar to untreated RECs. Vimentin expression levels in untreated RECs were consistent with a previous study [33], which showed that healthy inferior turbinates have lower expression of vimentin than nasal polyps.

In order to evaluate the potential of OE to prevent EMT, an EMT-induced model of RECs was developed by TGFβ1 induction (Fig. 2). Upon TGFβ1 induction, RECs showed reduction of cell proliferation (Fig. 4b) and changes in morphology from a cobblestone-like shape to a spindle shape (Fig. 3). In order to confirm whether those changes were an indication of EMT, the expression levels of vimentin and E-cadherin were investigated. Our results showed that RECs induced with TGFβ1 have lower expression of E-cadherin and higher expression of vimentin compared with controls, in line with the findings of several previous studies, which showed that, upon treatment with TGFβ1, epithelial cells, for example, Madin–Darby canine kidney (MDCK) cells displayed increased vimentin expression and reduced E-cadherin expression [25, 26].

TGFβ1 is known as an EMT inducer that predominantly targets Smad-dependent pathway activation [34]. Binding of TGFβ to TGFβ receptor will activate its downstream protein via a series of phosphorylation events of Smad 2 and Smad 3, which consequently form trimers with Smad 4. This complex is then translocated to the nucleus and will activate the transcription of target genes, for example, Snail, Zeb, FOXA1, PRX1 and Twist. These transcription factors will bind to the E-cadherin promoter, repress its transcription and simultaneously upregulate mesenchymal markers (vimentin) [35, 36].

In this study, the potential of OE to inhibit EMT when present together with an EMT induction factor was evaluated. Upon OE treatment of RECs undergoing TGFβ1-induced EMT, it was observed that OE helps to retain the cuboidal shape of RECs, hence preventing the effect of TGFβ1. However, after treatment with OE for 72 h, the vimentin expression of untreated RECs showed significant reduction compared with that of RECs undergoing TGFβ1-induced EMT. This might suggest that OE helps RECs to re-establish vimentin and E-cadherin expression; thus, OE has the potential to prevent EMT. There is less evidence on how and where *Olea europeae* targets the EMT signaling pathway. However, a previous study showed that treatment of OE inhibits the upregulation of SMAD4 and SNAIL2 (Slug), TCF4, VIM (Vimentin), FN (fibronectin) and SERPINE1 genes in a breast cancer cell line and MDCK cells [25]. Future work involving markers of the EMT signalling pathway looking at their expression could be beneficial to a greater understanding of the effects of OE on EMT.

## Conclusion

Our findings suggest that *Olea europeae* extract has the ability to prevent EMT by maintaining the epithelial phenotype. This proves that *Olea europeae* has the potential to modulate EMT and hence, can prevent persistent inflammation and tissue remodeling as in chronic rhinosinusitis. More studies using in vivo disease models are needed to confirm the utility of *Olea europeae* as an alternative treatment for airway diseases.

## Abbreviations

AEGM: Airway epithelial growth medium
DPBS: Dulbecco’s Phosphate-Buffered Saline
EMT: Epithelial-to mesenchymal transition
F12:DMEM: Dulbecco’s Modified Eagle Medium: Nutrient Mixture F-12
FBS: Fetal Bovine Serum
h: Hours
IC_50_: Inhibitory concentration
MDCK: Madin–Darby canine kidney
OE: Olive extract
PFA: Paraformaldehyde
RECs: Respiratory epithelial cells
SEM: Standard error of mean
TGFβ1: Transforming growth factor β1
